# Clinical Effectiveness of Hymenoptera Venom Immunotherapy: A Prospective Observational Multicenter Study of the European Academy of Allergology and Clinical Immunology Interest Group on Insect Venom Hypersensitivity

**DOI:** 10.1371/journal.pone.0063233

**Published:** 2013-05-20

**Authors:** Franziska Ruëff, Bernhard Przybilla, Maria Beatrice Biló, Ulrich Müller, Fabian Scheipl, Michael J. Seitz, Werner Aberer, Anna Bodzenta-Lukaszyk, Floriano Bonifazi, Paolo Campi, Ulf Darsow, Gabrielle Haeberli, Thomas Hawranek, Helmut Küchenhoff, Roland Lang, Oliviero Quercia, Norbert Reider, Peter Schmid-Grendelmeier, Maurizio Severino, Gunter Johannes Sturm, Regina Treudler, Brunello Wüthrich

**Affiliations:** 1 Department of Dermatology and Allergology, Ludwig-Maximilians-Universität, München, Germany; 2 Allergy Unit, Department of Internal Medicine, Allergy, Immunology and Respiratory Diseases, Ospedali Riuniti di Ancona, Azienda Ospedaliero-Universitaria, Ancona, Italy; 3 Allergiestation Medizinische Klinik, Spital Ziegler, Spitalnetz Bern, Switzerland; 4 Statistical Consulting Unit, Department of Statistics, Ludwig-Maximilians-Universität, München, Germany; 5 Department of Dermatology, Medical University of Graz, Graz, Austria; 6 Department of Allergology and Internal Medicine, Medical University of Bialystok, Bialystok, Poland; 7 Allergy Clinic, Nuovo Ospedale San Giovanni di Dio, Florence, Italy; 8 Department of Dermatology and Allergy Biederstein, Technische Universität München, and ZAUM – Center for Allergy and Environment, Munich, Germany; 9 Department of Dermatology, Paracelsus Medical University, Salzburg, Austria; 10 Dipartimento di Medicina Interna, Ospedale per gli Infermi, Faenza, Italy; 11 Department of Dermatology, Medical University of Innsbruck, Innsbruck, Austria; 12 Allergiestation, Dermatologische Klinik und Poliklinik, Universitätsspital Zürich, Zürich, Switzerland; 13 Klinik für Dermatologie, Venerologie und Allergologie, Universitätsklinikum Leipzig, Germany; University of Texas Health Science Center at San Antonio, United States of America

## Abstract

**Background:**

Treatment failure during venom immunotherapy (VIT) may be associated with a variety of risk factors.

**Objective:**

Our aim was to evaluate the association of baseline serum tryptase concentration (BTC) and of other parameters with the frequency of VIT failure during the maintenance phase.

**Methods:**

In this observational prospective multicenter study, we followed 357 patients with established honey bee or vespid venom allergy after the maintenance dose of VIT had been reached. In all patients, VIT effectiveness was either verified by sting challenge (n = 154) or patient self-reporting of the outcome of a field sting (n = 203). Data were collected on BTC, age, gender, preventive use of anti-allergic drugs (oral antihistamines and/or corticosteroids) right after a field sting, venom dose, antihypertensive medication, type of venom, side effects during VIT, severity of index sting reaction preceding VIT, and duration of VIT. Relative rates were calculated with generalized additive models.

**Results:**

22 patients (6.2%) developed generalized symptoms during sting challenge or after a field sting. A strong association between the frequency of VIT failure and BTC could be excluded. Due to wide confidence bands, however, weaker effects (odds ratios <3) of BTC were still possible, and were also suggested by a selective analysis of patients who had a sting challenge. The most important factor associated with VIT failure was a honey bee venom allergy. Preventive use of anti-allergic drugs may be associated with a higher protection rate.

**Interpretation:**

It is unlikely that an elevated BTC has a strong negative effect on the rate of treatment failures. The magnitude of the latter, however, may depend on the method of effectiveness assessment. Failure rate is higher in patients suffering from bee venom allergy.

## Introduction

About 10% of patients suffering from Hymenoptera venom allergy present with an elevated baseline tryptase concentration (BTC >11.4 µg/L) [1]. BTC is believed to represent the individual mast cell burden of a patient. To a large part, increased concentrations result from systemic mastocytosis or monoclonal mast cell activation syndrome [2]. Elevated BTC may also be found in patients with other mast cell diseases like chronic urticaria [3], uremic pruritus [4] or in patients with myeloid malignancies [2]. Apart from mastocytosis, mast cell number and life span may be chronically elevated in unselected allergic patients [5].

In allergic patients, it is unequivocally established that an increased BTC is a dominating risk factor for severe systemic reactions after a field sting by wasps or honey bees [1], [6]. During venom immunotherapy (VIT), the importance of BTC for severe side effects is less clear. A strong, independent association could only be shown in patients treated with wasp venom during the build-up phase [7]. After VIT, the importance of BTC for treatment failure is also controversial. Some consider BTC as an important predictor of the chance of VIT failure, and of the risk of relapse if VIT is stopped [8], whereas others were unable to find a strong association between a high BTC and the effectiveness of VIT [9]. For patients with systemic mastocytosis, in whom a particularly high BTC (>20.0 µg/l) is an important diagnostic criterion, some authors concluded that VIT may reduce the frequency of subsequent systemic allergic reactions to a clearly lesser extent than in allergic patients not presenting with clonal mast cell diseases [10], [11]. Others, however, claim that VIT is safe and effective in patients with mastocytosis [12].

It was the aim of the present prospective international multicenter study to determine the importance of BTC and of other suspected risk factors for VIT failure during the maintenance phase of VIT. Furthermore, since the precise magnitude of the treatment effect is still unknown, the present study also wanted to examine the rate of VIT failure in an unselected patient cohort suffering from Hymenoptera venom allergy. The first and the second part of that study, which examined risk factors for severe anaphylactic reactions after a field sting which preceded VIT, or for severe side effects during the build-up phase of VIT, were published recently [1], [7].

## Methods

### Study Design

The Tryptase in Hymenoptera Venom Allergy (TIHVA) study of the Interest Group on Insect Venom Hypersensitivity of the European Academy of Allergology and Clinical Immunology (EAACI) is a prospective observational cohort study, which was performed in 14 European clinics specialized on the diagnosis and treatment of allergic diseases. In patients suffering from Hymenoptera venom allergy, we had evaluated risk factors for severe systemic reactions after a field sting (before VIT) and during the build-up phase of VIT (part I and II of the study) [1], [7]. In part III of the study, we now present data on the therapeutic effectiveness of VIT in a patient subgroup examined during the maintenance phase of VIT. Design of the TIHVA study, patient enrolment, diagnostic procedures, laboratory tests, data accuracy and characteristics of the core population have been presented in detail in previous publications [1], [7].

For the present study, we analyzed those patients in whom the effectiveness of VIT could be assessed (either by in-hospital sting challenge or by patient-reported reactions to accidental field stings). Patients were excluded if they had not undergone a sting challenge or if they had not sustained a sting by the culprit insect during follow up. Patients who had sustained a field sting and who were uncertain about the species of the stinging insect, or in whom symptoms were equivocal, or whose history could not precisely be obtained, were also excluded from the analysis. We also did not incorporate patients into the study who had had a field sting by the culprit insect during the build-up phase of VIT. Patients, who were allergic to both bee and wasp venom, could not be evaluated in the present study. Absence of a double allergy was a prerequisite for being enrolled in the core population of this long-term project [1].

### Venom Immunotherapy

For the majority of subjects, the maintenance dose was 100 µg. In selected high risk patients (bee keepers or other patients with a particularly high risk of insect exposure) and in some of the patients who had experienced severe side effects during the maintenance phase, a maintenance dose of 200 µg was used. Indications for using a higher venom dose were not specified.

The method and date of effectiveness assessment (in-hospital sting challenge or patient-reported reactions to accidental field stings) was left to the discretion of the treating study centre. Sting challenges were performed according to European guidelines [13] which also indicate specific contraindications such as unstable internal diseases (asthma, cardiac disease), or pregnancy. Emergency treatment followed specific guidelines. Even in the absence of hemodynamic symptoms, all cases developing cutaneous symptoms during sting challenge were treated immediately.

### Patient Management during the Maintenance Phase of VIT

At regular time intervals, all patients were seen as outpatients for therapy continuation (3 to 5 years after the end of the build-up phase). Patients were asked for symptoms which might have occurred after a field sting by the culprit insect. For treatment of symptoms in the event of being stung by the culprit insect, all patients had been provided with an emergency kit including an H_1_-blocking high-dose antihistamine, a corticosteroid and an adrenaline auto-injector. In addition, patients with airway symptoms received a bronchodilator. Patients had been advised to immediately take antihistamines and corticosteroids after a field sting, and before the onset of symptoms [14], [15]; adrenaline auto-injectors should only be used in case of a systemic reaction.

In addition to emergency treatments, preventative measures were provided including education (avoidance advice) on how to avoid bee and/or wasp stings, and on how to recognise the early symptoms of anaphylaxis.

### Baseline and Test Variables

Besides age (at the time of the sting challenge, or of the first eventful or the last uneventful field sting) and gender, we recorded preventive use of an emergency medication after a field sting (use of oral antihistamines or of antihistamines and corticosteroids before the appearance of clinical symptoms), venom dose during maintenance therapy, the type of antihypertensive medication, which was taken during the sting challenge or field sting and the type of venom used for therapy. We also recorded the frequency of systemic allergic reactions during the build-up or maintenance phase of VIT, and the severity grade (according to Ring [16]) of the most severe sting reaction prior to VIT. We furthermore, collected information on the time interval between the end of the build-up phase of VIT and the day of the sting challenge, or of the first eventful or the last uneventful field sting. We also documented whether effectiveness had been assessed by sting challenge or patient-reported reactions. When taking the patient’s history, particular attention was paid to the order of events (e.g., medication before or after the onset of symptoms). Test variable was the baseline serum tryptase concentration (BTC).

End point of the present analysis was an objective systemic reaction during an in-hospital sting challenge or after a field sting by the culprit insect. Objective systemic reactions included itching and urticaria (but not itching alone), flush and hemodynamic shock. At least one of these symptoms must have occurred to diagnose a systemic reaction. Three conditions were defined as being compatible with signs of a hemodynamic shock: a) heart rate>systolic blood pressure when recorded during sting challenge, b) heart rate>systolic blood pressure registered by an emergency physician after a field sting, and c) temporary loss of consciousness registered by the patient, by bystanders or by an emergency physician after a field sting (assuming that low blood pressure leads to unconsciousness). In the absence of hemodynamic shock, itching/urticaria or flush, we considered dizziness or light-headedness an uncertain reaction. Corresponding patients were excluded from the analysis. If a patient had already experienced systemic symptoms of any kind, and had subsequently taken anti-allergic drugs, s/he was counted among those in whom VIT had failed, and was not counted among patients who had prophylactically taken anti-allergic drugs.

Patients, who exclusively reported shortness of breath, coughing, or anxiety reactions, and who had recovered without taking any emergency medication, were thought to have tolerated the venom. Reason for the latter was the finding that, in patients allergic to Hymenoptera venom, a self-limiting systemic allergic reaction which is exclusively confined to respiratory symptoms is an extraordinarily rare event. In honey bee/wasp venom allergy, exclusive and self-limiting respiratory symptoms rather result from an unspecific psycho-vegetative reaction [17].

### Statistics

Categorical variables were expressed as percentage, metric variables as median and interquartile range. Selective Comparisons between patient groups were made by Fisher’s exact test for binary variables.

To estimate smooth (non-linear) effects of metric variables, we used penalized regression splines. Smoothing parameters were selected by the generalized cross validation criterion. Because of low data density, we penalized the effect of binary covariates [18]. Covariate-adjusted effects of baseline tryptase concentration (BTC) on the effectiveness of VIT were evaluated by multiple logistic regression models, which combined separate effects of all individual confounding variables (generalized additive models (GAMs), [19]). GAMs were estimated using an R package [20].

For the dependent variable we used the best subset selection method to identify a separate starting model which did not include the variable BTC [8]. A random effect, however, was included to adjust for study centre. Model performance was assessed using areas under the curve (AUCs) derived from receiver operating characteristic (ROC) analyses of the model [21]. To identify the best model we used a stratified 5-fold cross validation with 5 rounds. To test the tryptase effect, we then added the variable “baseline tryptase concentration” to the starting GAM with the largest AUC thereby creating the final GAM. Parametric and semi-parametric effects for tryptase were estimated.

To examine whether the effect of BTC depended on the type of venom, we used the final GAM to tested interactions between venom type and BTC. Furthermore, since early prophylactic use of anti-allergic drugs could have prevented development of symptoms after a field sting by the culprit insect, we re-estimated the final GAM using data which did not include corresponding patients to assess the stability of the estimated effects. We also constructed another final GAM using only data from patients in whom VIT effectiveness had been assessed by sting challenge.

## Results

### Clinical Characteristics of Patients in whom Effectiveness of VIT could be Assessed

The Tryptase in Hymenoptera Venom Allergy study of the Interest Group on Insect Venom Hypersensitivity of the EAACI recently reported outcomes of 680 patients during the build-up phase of VIT [7]. Effectiveness of VIT could be assessed in 357 patients during the maintenance phase. The majority of patients were male (59.1%) and were suffering from wasp venom allergy (74.8%). Median age was 45 years (33–59 years). 32 (9.0%) of the patients had a BTC >11.4 µg/L (maximum 101.0 µg/L), and 7 (2.0%) >20 µg/L. 27.2% of the patients had had a grade III or IV reaction at the index field sting which preceded immunotherapy. During the build-up or maintenance phase of immunotherapy, systemic allergic reactions had occurred in 8.4% of the patients.

14 patients (3.9%) had been treated with a maintenance venom dose of 200 µg. 10 of these patients had been thought to be at a particularly high risk and had, therefore, received the 200 µg dose. There were only four patients in whom we had started with a 100 µg dose, and who subsequently were switched to the 200 µg dose after systemic allergic reactions had occurred during the maintenance phase of VIT.

No follow up was possible in 323 patients. Compared to the study group (n = 357 patients), excluded patients were younger (age 39.5 years (28–51 years), p<0.001) and were less often allergic to wasp venom (63.2%, p = 0.001). The frequency of male patients (55.3%, p = 0.321) and of patients presenting with an increased BTC (>11.4 µg/l) (10.8%, p = 0.795) was, however, comparable. Of those 323 patients, 303 had to be excluded because of a missing field sting/sting challenge; in 4 patients there was a significant uncertainty regarding the stinging insect, 13 were stung be the presumably wrong insect and 3 patients presented with equivocal symptoms of their post-sting reaction. Of patients in whom the identity of the stinging insect was uncertain or not relevant, none had a systemic allergic reaction after the field sting.

Of patients included into the present study, 154 underwent an in-hospital sting challenge (43.1%); the remainder of the patients (n = 203) had a field sting by the culprit insect. Median time elapsing between the end of build-up and sting challenge/field sting by culprit insect was 17 months (10–34 months). 14.3% of the patients took various types of antihypertensive medication at the time of the sting challenge/field sting. 2.5% of patients were on beta-blocker therapy, and 4.2% on Angiotensin converting enzyme (ACE)-inhibitor therapy.

29 patients who were stung by the culprit insect (and who did not have a sting challenge) used an emergency medication. These patients exclusively took oral antihistamines or antihistamines and corticosteroids with intent to prevent allergic reactions. In patients assessed by self reporting of field sting reactions (n = 203), preventive use of antihistamines or antihistamines and corticosteroids was only observed in a minority of these 203 patients (14.3%). Of 5 patients who had been stung by the culprit insect and who simultaneously presented with a BTC >20 µg/L, only one took oral antihistamines and corticosteroids right after the sting. None of the 29 patients who prophylactically took anti-allergic drugs had allergic reactions after a field sting. On the other hand, generalized reactions developed in 8.1% of the patients (14 of 174 patients) who had not used an emergency kit after a field sting (p = 0.107). Patient groups using or not using an emergency medication after a field sting differed slightly: Thus, 20.6% of all women, but only 11.1% of all men used such medications (p = 0.089). Parameter values of all other variables were not significantly different.

In the whole cohort, 22 patients (6.2%) required an emergency intervention during an in-hospital sting challenge or developed, after a field sting by the culprit insect, generalized symptoms. This failure rate is based on the assumption that preventive use of antihistamines or antihistamines and corticosteroids would have in fact been unnecessary because of a sufficient protection. However, we cannot exclude the possibility that a prophylactic anti-allergic medication was effective in preventing a generalized systemic reaction which would have been observed without such a therapy. Assuming such a bias in patients, who had taken antihistamines or antihistamines and corticosteroids after a sting, the frequency of VIT failure would be higher maximally amounting to 7.0%.

### Risk Factors for VIT Failure

Unadjusted results are presented in Table 1. Without considering confounders, there was no evidence that the frequency of VIT failure varied by the venom dose used (100 µg: 5.8%, 200 µg: 14.3%, p = 0.211), or by the method used to assess effectiveness (failure rates according to self-reporting after a field sting: 6.9%, according to sting challenge: 5.2%, p = 0.660).

**Table 1:** Distribution of therapy failures during the maintenance phase of VIT with respect to baseline parameters.

Variable		Emergency intervention (sting challenge)or generalized symptoms (field sting)		p value
		No (n = 335)	Yes (n = 22)	
Gender	male	199 (94%)	12 (6%)	0.407
	female	136 (93%)	10 (7%)	
Highest degree of index sting reaction preceding VIT	I or II	243 (93%)	17 (7%)	0.420
	III or IV	92 (95%)	5 (5%)	
Type of venom	wasp	255 (96%)	12 (4%)	0.027
	honey bee	80 (89%)	10 (11%)	
Venom dose (µg) during maintenance therapy	100	323 (94%)	20 (6%)	0.211
	200	12 (86%)	2 (14%)	
Side effects during build-up or maintenance phase	yes	26 (87%)	4 (13%)	0.102
	no	309 (94%)	18 (6%)	
ACE-inhibitor medication at sting challenge/field sting	yes	15 (100%)	0 (0%)	0.378
	no	320 (94%)	22 (6%)	
Beta-blocker medication at sting challenge/field sting	yes	9 (100%)	0 (0%)	0.560
	no	326 (94%)	22 (6%)	
Any antihypertensive medication at sting challenge/field sting	yes	39 (98%)	1 (2%)	0.148
	no	296 (93%)	21 (7%)	
Verification of VIT effectiveness by sting challenge	yes	146 (95%)	8 (5%)	0.333
	no	189 (93%)	14 (7%)	
Preventive use of oral antihistamines or antihistaminesand corticosteroids after the field sting	yes	29 (100%)	0 (0%)	0.145
	no	306 (93%)	22 (7%)	
Age (years) at sting challenge/field sting according to median	<45	172 (93%)	12 (7%)	0.473
	≥45	163 (94%)	10 (6%)	
Time interval (months) between the end of build up andsting challenge/field sting according to median	<17	172 (92%)	14 (8%)	0.185
	≥17	163 (95%)	8 (5%)	
BTC (µg/l) according to normal value	≤11.4	304 (93%)	21 (7%)	0.393
	>11.4	31 (93%)	1 (3%)	

Associations are shown between clinical, demographic and therapeutic parameters and the need for an emergency intervention during an in-hospital sting challenge or, after a field sting by the culprit insect, the development of any type of generalized symptom.

VIT failure was observed more often in patients receiving a honey bee VIT (11% vs. 4% in patients receiving a vespid VIT). The difference was even more pronounced in those patients in whom VIT effectiveness could be assessed by an in-hospital sting challenge (19% vs. 1%, p<0.001). There was also a tendency for a lower protection rate in patients, who had had a systemic allergic reaction during the build up or maintenance phase of VIT (13% vs. 6%, p = 0.102), whereas preventive use of oral antihistamines or antihistamines and corticosteroids appeared to reduce the frequency of generalized symptoms after a field sting (see above). An increased BTC (>11.4 µg/L) did not increase the frequency of VIT failure (3% vs. 7% in patients with a BTC ≤11.4 µg/L, p = 0.393).

When selecting variables for the starting GAM (which did not include the variable BTC), a random study centre effect was retained in the model. Consequently, the final starting model was adjusted for such an effect. According to AUC values, the best model was that which included the variables “therapy with honey bee venom”, “assessment of effectiveness by sting challenge”, “ACE-inhibitor medication at sting challenge/field sting” and “ preventive use of oral antihistamines or antihistamines and corticosteroids after the field sting” (AUC = 0.7182).

**Figure 1:**
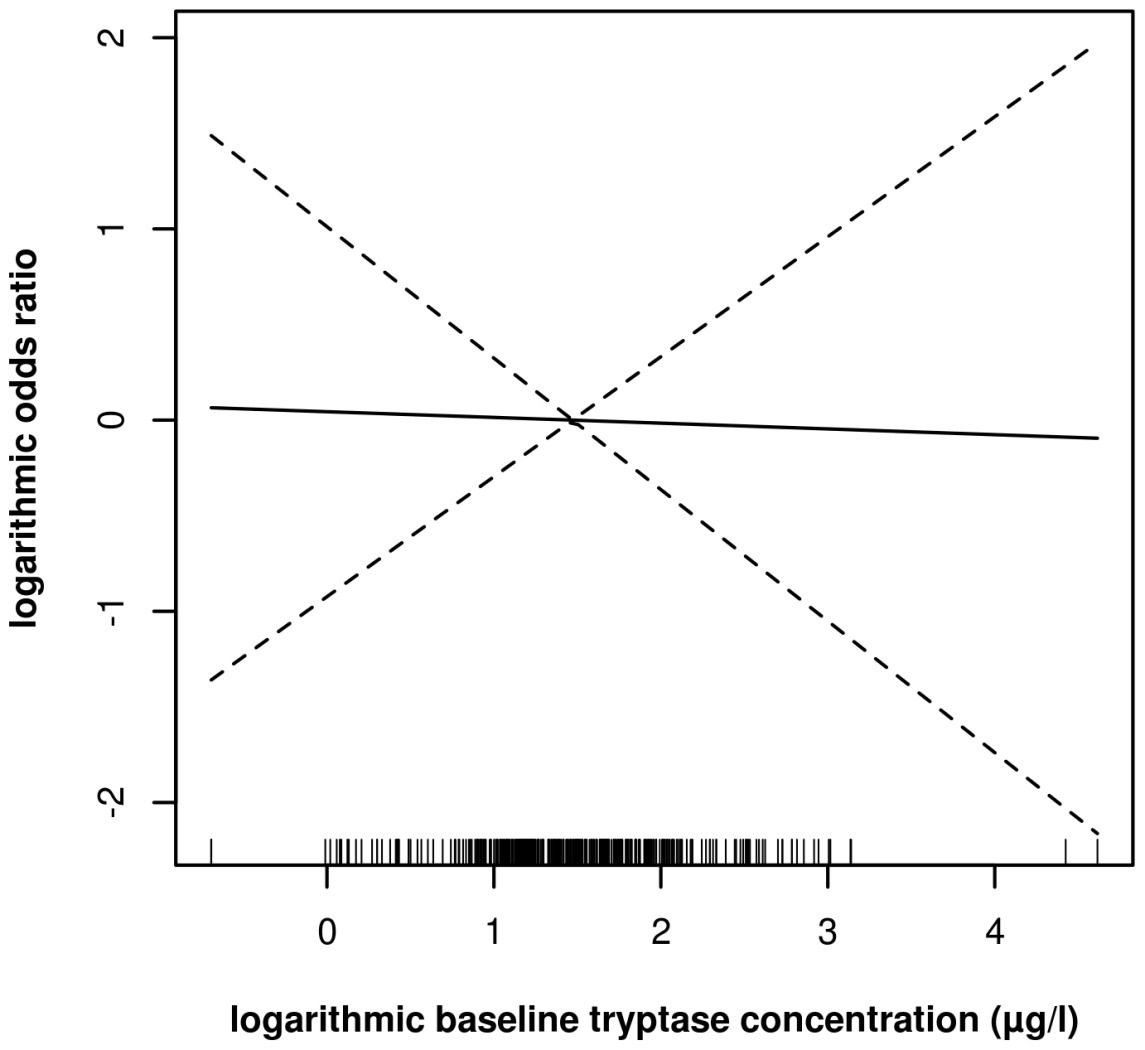
Receiver operating characteristic (ROC) curve for the final multiple logistic regression model predicting the risk to need an emergency intervention during sting challenge or to develop generalized symptoms after a field sting. Models were tested without including the effect of BTC, or with including a smoothed or a linear effect. Corresponding areas under the curve were 0.7449, 0.7084 or 0.7240.

**Figure 2:**
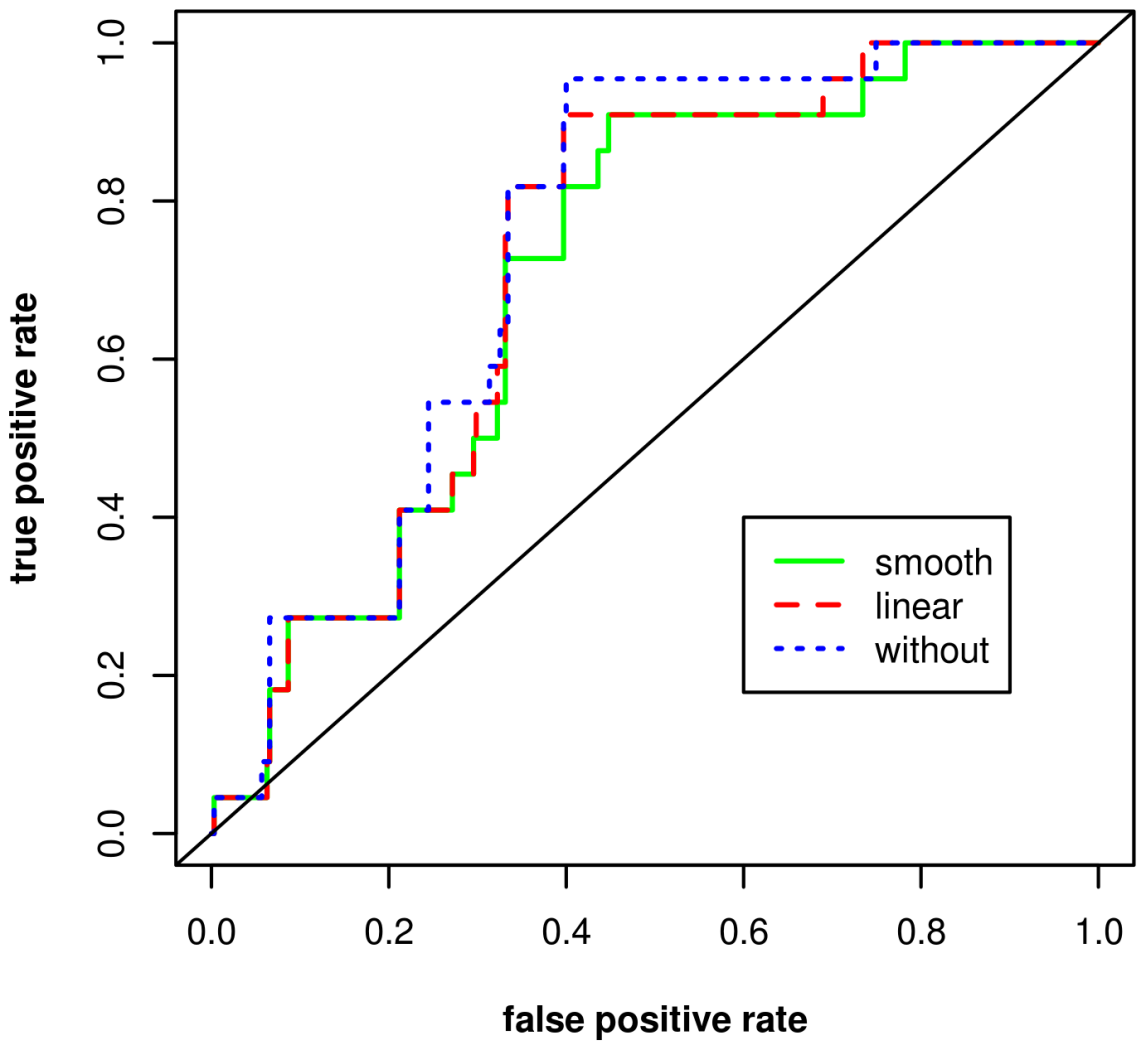
Smooth function and pointwise 95% confidence bands (dashed lines) for the effect of baseline tryptase concentration on the risk to need an emergency intervention during sting challenge or to develop generalized symptoms after a field sting (final multivariate generalized additive model). Odds ratios are referred to those of the median of tryptase concentration. The odds ratio of the latter has been set at 1.

After adjustment for the other confounders, we did not observe a significant association between BTC and the risk for VIT failure (Figure 1). Irrespective from the type of effect examined (smooth or linear), incorporation of BTC also did not increase the AUC of the starting model (Figure 2). The width of the confidence bands (Figure 1), however, allowed us to rule out major effects of BTC (e.g., in comparison to a reference patient with a BTC of 4.3 µg/L (the sample mean), the odds ratio for a patient with a BTC of 20 µg/L is unlikely to be higher than 2.7).

Inclusion of the variable BTC into the starting model did not appreciably change the importance of other confounders (Table 2). Thus, a therapy with honey bee venom remained a potential predictor for VIT failure, whereas use of an emergency medication after the field sting, and assessment of effectiveness by sting challenge presumably had lowered this risk. The latter finding is remarkable, since in the unadjusted analysis we could not find an association between the type of effectiveness assessment and VIT failure emphasizing the importance of considering other confounders when estimating the predictive power of an individual parameter. The comparably small number of patients in whom treatment failed, however, prevented the identification of clearly significant effects (p-values between 0.05 and 0.1).

**Table 2:** Results of the final generalized additive model for the risk to need an emergency intervention during an in-hospital sting challenge or, after a field sting by the culprit insect, to develop any type of generalized symptom.

Variable	p value	odds ratio	95% confidence interval	
Therapy with honey bee venom	0.100	2.209	0.860	5.675
Verification of VIT effectiveness by sting challenge	0.070	0.344	0.109	1.091
ACE-inhibitor medication	0.495	0.461	0.050	4.265
Preventive use of oral antihistamines or antihistamines and corticosteroids after the field sting	0.101	0.203	0.030	1.362

Those variables are shown, which were selected according to the modeling procedure. P-values and widths of confidence intervals are biased downwards due to the effect of subset selection.

The variable “ACE-inhibitor medication at sting challenge/field sting” was also retained in the final model. However, the precise importance of this variable for risk prediction remains unknown, because of the large uncertainty associated with its estimated effect. Finally, several other variables were not selected for the final model by the AUC based algorithm indicating the prognostic unimportance of age, gender, venom dose during maintenance therapy, other types of antihypertensive medication, frequency of systemic allergic reactions during the build-up or maintenance phase of VIT, severity of the most severe sting reaction prior to VIT, and duration of VIT.

There was also no evidence that the effect of BTC varied between wasp and honey bee VIT. After including interactions between venom type and BTC into an extended model, the specific association between venom type and BTC was not significant (p>0.5). Furthermore, incorporation of different types of BTC effects (linear or smooth) did not increase the AUC of the extended model.

Since we found evidence that preventive use of antihistamines or antihistamines and corticosteroids might have prevented subsequent allergic reactions (thereby possibly obscuring the assessment of VIT effectiveness), the statistical analysis was repeated after exclusion of patients having used this self-medication. Corresponding models and odds ratios, however, remained virtually unchanged.

Since results obtained by self-reporting of field sting reactions might be biased, and since we found evidence that verification of VIT effectiveness depends on the method used, we performed an additional statistical analysis using only data from patients who had a sting challenge to assess VIT effectiveness. In that subgroup analysis, estimation of the variable “therapy with honey bee venom” yielded an odds ratio of 28.1 (95% confidence interval 3.65–217, p = 0.001), and estimation of the variable “BTC” an odds ratio of 2.18 (after a logarithmic transformation; 95% confidence interval 0.68–7.10, p = 0.183). These estimates were much more pronounced than those based on the full data set.

## Discussion

Our study is the largest to evaluate the importance of BTC in the serum and of a variety of other suspected risk factors for treatment failure during the maintenance phase of VIT. In our study, VIT failure was either defined as an objective generalized symptom during an in-hospital sting challenge or as a systemic reaction after a field sting by the culprit insect. We found that the rate of VIT failure may vary between 6.2% and 7.0% (if it is assumed that a systemic allergic reaction would have occurred in all patients who had taken preventive anti-allergic drugs).

The effectiveness of VIT is beyond doubt. Two systematic reviews [22], [23] and one meta-analysis [24] have concluded that VIT is effective in preventing future systemic reactions to venom in patients with hymenoptera venom allergy. The magnitude of the effect, however, is highly controversial. According to numerous randomized, quasi experimental or non-comparative studies, the reported rates of VIT failure range between 0 and 36% [25]. The quality of these studies is mostly poor due to small sample size, and all studies are single centre studies reflecting the experience of a single institution thereby preventing a generalization of the results. Furthermore, there is substantial heterogeneity in terms of differences in venom extracts and concentrations, differences in administration methods (updosing and/or maintenance programs, type and length of treatment), differences in the type of effectiveness assessment, in timing of re-stings and in the proportion of patients being re-stung. Hockenhull et al [25] recently pooled data from nine randomized and non-randomized studies which had used Pharmalgen® for VIT. In these studies, VIT effectiveness had either been assessed by sting provocation or by accidental field stings. Furthermore, there was no evidence that field sting reactions could have been obscured by an anti allergic emergency pre-treatment. The authors found a rate of VIT failure of 6.5% (22 systemic reactions in 337 patients). This rate corresponds closely to our findings and suggests that, in unselected patients and despite the above limitations, the effectiveness of VIT is excellent and highly reproducible.

The key finding of our study is that there was no strong association between BTC and the frequency of treatment failure during the maintenance phase of VIT. Due to the width of the confidence bands, however, smaller effects of BTC cannot be excluded. It has recently been speculated that there might be negative effects of an elevated BTC/mastocytosis on the success rate of VIT [10]. A systematic review pooled data from seven observational studies examining VIT effectiveness in mastocytosis. The authors found that 28% of the treated patients (23 of 82 patients) had a systemic reaction to a re-sting [10]. This failure rate is almost five times higher than the average failure rate of 6% found in unselected cohorts (including the one in the present study). According to our data, however, the magnitude of the BTC/mastocytosis effect might have been overestimated in that analysis, which could not adjust results to potential confounders.

The size of our cohort would have been sufficient to detect a more than 4-fold increase of the rate of VIT failure due to an elevated BTC (>11.4 µg/l). Assuming that about 10% of patients present with an increased BTC (>11.4 µg/l), and assuming further that the probability of VIT failure is about 5% for patients, who have a normal BTC, it may be calculated that a study would have to analyse at least 331 patients to detect such an increase in frequency (two-sided type I error, 5 percent; power, 80 percent). Our data provide some evidence, however, that weaker effects of BTC might indeed exist. A subgroup analysis in patients in whom we had used sting challenge to asses VIT effectiveness revealed such a weak BTC effect (OR 2.18 for a 2.7 fold increase of BTC) which was retained in the final statistical model and which, for reasons discussed below, might be more reliable than the effect estimated for the whole cohort. Consequently, our findings suggest that a minority of patients (presumably those presenting with a particularly high BTC) may not benefit from a standard VIT to the same extent as patients with a normal BTC.

Several other conclusions may be derived from our results. In accordance with numerous other studies, bee venom allergy was an independent predictor for VIT failure. A systematic review found that only 0–9% of patients allergic to wasp venom, but about 20% of bee venom allergic patients still reacted to a sting challenge with the culprit insect [11]. It is also established that immunotherapy with bee venom is a major risk factor for severe side effects during the build-up phase of VIT [7], [11]. On the other hand, we and others have shown that, before VIT, honey bee venom allergy is an important predictor for a lower risk of a severe systemic reaction after a field sting [1], [26], [27]. Consequently, the worse results associated with honey bee venom immunotherapy must be attributed to the treatment itself. Thus far, it is entirely unclear why a treatment with honey bee venom is associated with a higher rate of side effects and treatment failures. We have recently speculated that this phenomenon relates to the amount of venom dose applied during therapy. In honey bee and wasp VIT the total number of therapeutic injections is the same. In comparison to wasp VIT and to the amount of wasp venom emitted during a field sting, patients receiving a honey bee venom immunotherapy are, however, exposed to a significantly greater number of injections, which provide subclinical amounts of venom thereby possibly favouring pro-allergic reactions during therapy, and treatment failures [7]. Conversely, it is also possible that wasp VIT is more effective than bee VIT because there are marked differences in the amount of venom emitted during a field sting (honey bee: 50 to 100 µg; wasp: 3 to 5 µg), whereas the amount of venom applied during the maintenance phase of VIT is identical. Therefore, compared to field sting conditions, patients allergic to wasp venom receive a much greater dose possibly resulting in a better protection.

Another interesting finding of our study was that the method which had been used to assess effectiveness correlated with the chance of VIT failure. VIT seemed to be less effective when evaluated by patient self-reporting (systemic allergic reactions after a field sting) than when evaluated by in-hospital sting challenge. Thus, the adjusted frequency of systemic allergic reactions was lower during sting challenge than after a field sting. Differences between those two methods used to provoke an allergic reaction are well established and have been demonstrated again recently by studies assessing the effectiveness of VIT in patients with mastocytosis [10], [13].

To explain these divergent results two different hypotheses have been put forward. The first assumes that the risk for severe allergic reactions is greater during normal life than when being deliberately tested in well-prepared patients in a highly artificial environment excluding those who have contraindications for sting challenge [13]. In such a setting this bias would artificially reduce the frequency of VIT failures. The second hypothesis focuses on the historical assessment of preceding symptoms. Within such a procedure a bias may exist affecting the reliability of information obtained by self-reporting after a field sting. There are numerous misconceptions related to a patient’s comprehension, recall, evaluation and expression [28], [29]. Despite VIT, a new sting may be preconceived by the patient as a risk factor of future severe allergic reactions making him inflate the importance of post-sting symptoms. Thus, subjective symptoms after a field sting can be described as severe by patients but can be viewed as subjective and not significant during sting challenge. Such a mechanism would artificially increase the frequency of VIT failures after a field sting. The relative importance of those two hypothesis is unknown but allergists tend to favour the second one [30].

Three other aspects of our results deserve a specific comment. When analyzing the use of self medication after a field sting by the culprit insect, we found an association with the rate of VIT failure (Table 2). Although significance was marginal, this effect was retained in the final statistical model emphasizing the prognostic importance of this variable. Preventive use of oral antihistamines or antihistamines and corticosteroids appeared to decrease the risk of a systemic allergic reaction thereby supporting corresponding guideline and practice parameter recommendations [8], [11], [31]. It should be noted, however, that this finding is also subject to a recall bias. Patients feeling reassured by self-medication might have been less likely to report symptoms.

Only about 14% of the patients used oral antihistamines or antihistamines and corticosteroids to prevent allergic reactions after a field sting. This finding precisely corresponds to the depressingly low frequency (about 14%) with which oral antiallergic drugs are taken after unselected severe allergic reactions in the community of German speaking countries [32]. These low frequencies reveal an extraordinarily poor patient compliance and underscore the need for an intensified patient education and training.

Finally, we found no evidence that side effects during the build-up or maintenance phase correlated with treatment effectiveness. During VIT, the strongest predictor for severe side effects is a therapy with honey bee venom [7]. Since the latter variable is also associated with the chance of VIT failure, it is likely that the importance of the type of venom for treatment success outweighed the importance of side effects during VIT.

There was also no association between the duration of VIT and the frequency of VIT failure. It is commonly believed that the length of VIT correlates with the protection rate. A recent study, however, suggested that in the majority of allergic patients VIT is effective within a week after the maintenance dose has been achieved [33]. In most of the patients in our study, however, the effectiveness of VIT could be only assessed between months 10 and 34 of maintenance therapy. It appears that at least during this time span the protection rate largely remains unchanged.

### Strengths and Limitations of the Study

The major strength of this study is the large number of patients, and the prospective und multicentre design allowing a generalization of obtained findings. The study, however, is also subject to several limitations. Only about half of the original patient cohort which had had a VIT [7] was evaluable in terms of effectiveness assessment (either by sting challenge or self reporting of outcomes after a field sting). This might represent a selection bias, particularly in view of the fact that, compared to patients in the present study, excluded patients differed in terms of age and frequency of bee/wasp venom allergy. Furthermore, self reporting implies errors concerning the correct identification of the insect involved in a field sting, the correct reporting of symptoms, and the true amount of venom delivered by the sting. In addition, the small number of events (VIT failures) prevented a thorough analysis of BTC and of a variety of other variables possibly important for the protection rate. Finally, it is also possible that a portion of the absent effect of BTC was due to an analytical error. A certain, albeit small number of patients might have presented with heterophilic antibodies which may falsely increase BTC [34], [35].
